# The host micro-RNA cfa-miR-346 is induced in canine leishmaniasis

**DOI:** 10.1186/s12917-022-03359-5

**Published:** 2022-06-27

**Authors:** Gloria Buffi, Aurora Diotallevi, Marcello Ceccarelli, Federica Bruno, Germano Castelli, Fabrizio Vitale, Mauro Magnani, Luca Galluzzi

**Affiliations:** 1 Department of Biomolecular Sciences, Section of Biotechnology, University of Urbino Carlo Bo, via Arco d’Augusto 2, 61032 Fano, PU Italy; 2 Centro di Referenza Nazionale Per le Leishmaniosi (C.Re.Na.L.), OIE Leishmania Reference Laboratory, Istituto Zooprofilattico Sperimentale della Sicilia A. Mirri, via G. Marinuzzi 3, 90129 Palermo, PA Italy

**Keywords:** mir-346, *Leishmania* spp., DH82, ER stress, Canine leishmaniasis

## Abstract

**Background:**

Leishmaniases are a group of anthropo-zoonotic parasitic diseases caused by a protozoan of the *Leishmania* genus, affecting both humans and other vertebrates, including dogs. *L. infantum* is responsible for the visceral and occasionally cutaneous form of the disease in humans and canine leishmaniasis. Previously, we have shown that *L. infantum* induces a mild but significant increase in endoplasmic reticulum (ER) stress expression markers to promote parasites survival in human and murine infected macrophages. Moreover, we demonstrated that the miRNA hsa-miR-346, induced by the UPR-activated transcription factor sXBP1, was significantly upregulated in human macrophages infected with different *L. infantum* strains. However, the ER stress response in infected dogs, which represent an important reservoir for *Leishmania* parasite, was described once recently, whereas the miR-346 expression was not reported before. Therefore, this study aimed to investigate these pathways in the canine macrophage-like cell line DH82 infected by *Leishmania* spp. and to evaluate the presence of cfa-miR-346 in plasma of non-infected and infected dogs.

The DH82 cells were infected with *L. infantum* and *L. braziliensis* parasites and the expression of cfa-mir-346 and several ER stress markers was evaluated by quantitative PCR (qPCR) at different time points. Furthermore, the cfa-miR-346 was monitored in plasma collected from non-infected dogs (*n* = 11) and dogs naturally infected by *L. infantum* (*n* = 18).

**Results:**

The results in DH82 cells showed that cfa-mir-346 was induced at both 24 h and 48 h post-infection with all *Leishmania* strains but not with tunicamycin, accounting for a mechanism of induction independent from sXBP1, unlike what was previously observed in human cell lines. Moreover, the cfa-miR-346 expression analysis on plasma revealed a significant increase in infected dogs compared to non-infected dogs.

**Conclusions:**

Here for the first time, we report the upregulation of cfa-miR-346 induced by *Leishmania* infection in canine macrophage-like cells and plasma samples of naturally infected dogs. According to our results, the cfa-miR-346 appears to be linked to infection, and understanding its role and identifying its target genes could contribute to elucidate the mechanisms underlying the host–pathogen interaction in leishmaniasis.

## Background

Leishmaniasis includes a group of anthropo-zoonotic diseases caused by a protozoan belonging to the *Leishmania* genus, which can have different hosts, like humans, dogs, or wild animals [1, 2]. According to WHO data, leishmaniasis shows a worldwide distribution, involving at least 98 countries, with the highest number of cases in developing countries [3]. Italy, in particular the southern regions (including Sicily and Sardinia), is a country highly endemic for leishmaniasis caused by *Leishmania* (*L*.) *infantum*, although cases of canine leishmaniasis (CanL) and human leishmaniasis are largely underestimated due to the lack of notification of new cases, and the difficulty in diagnosing or misdiagnosis [4, 5]. Although a subclinical or self-limiting form was described, CanL represents a very critical problem in veterinary medicine, because the disease can be severe and with fatal exitus [6].

*Leishmania* (*L*.) *infantum* (syn. *L.* (*L*.) *chagasi* in South America) is responsible for both VL and CL in humans [7], while in dogs is the most important aetiological agent of CanL in the Mediterranean Basin, Central America, South America and parts of Asia [8].

*Leishmania* is a dimorphic parasite that develops as promastigotes in the midgut of the sand-fly and as intracellular amastigotes in phagocytic cells, mainly macrophages, of the vertebrate host. *Leishmania* promastigotes enter the host macrophages, where they survive and replicate within the phagolysosomal environment, subverting the innate immune response and metabolic pathways of the host [9, 10]. Among different mechanisms activated by *L. (L.) infantum* to survive and spread the infection in the host cell, we previously reported the mild induction of the Unfolded Protein Response (UPR) in human monocytic cell lines (U937 and THP-1) and murine primary macrophages [11]. The mild UPR could represent a common pathogenic mechanism among the different species of *Leishmania* (*Leishmania*), also considering the role of X-box binding protein 1 (XBP1) in the *L. amazonensis* infection highlighted by Dias Teixeira *et al*. [12]. The unconventional splicing of XBP1 mRNA, induced by the endoplasmic reticulum (ER) transmembrane protein IRE1, originates a spliced mRNA (sXBP1) encoding an active transcription factor that leads to the expression of chaperones, proinflammatory cytokines [13], and genes involved in the autophagic response [14]. Few years ago, Bartoszewski *et al*. showed that, during ER stress, sXBP1 can induce the expression of miR-346 in both human and murine cells, whose target genes include the major histocompatibility complex (MHC) class I gene products and interferon-induced genes [15]. We recently investigated the expression of mir-346 in a human cell infection model, showing the significant induction of miR-346, in both U937 and THP-1 human cell lines following ER stress elicited by infection with four different strains of *L.* (*L.*) *infantum* and a human clinical isolate of *L*. (*V*.) *braziliensis* [16].

MicroRNAs have gained more and more relevance in the last years in the context of host–pathogen interaction. In fact, it is known that many intracellular parasites are able to modify the miRNA expression profiles of host cells [17]. In particular, in the context of CanL, Bragato *et al*. reported a differential modulation in the expression of miR-150, miR-451, miR-192, miR-194, and miR-371 in peripheral blood mononuclear cell (PBMC) of symptomatic dogs naturally infected with *L. infantum*. These miRNAs can target genes involved in the immune response and pathogenesis, such as NF-κB, TNF-α, CD80, and IFN-γ [18].

In this study, we investigated the UPR and the induction of *Canis lupus familiaris* cfa-miR-346 in a canine macrophage-like cell line (DH82) infected with *Leishmania* spp. Furthermore, the presence of cfa-miR-346 in the plasma of non-infected and infected dogs and its potential role as a marker of infection was analyzed.

## Results

### Tunicamycin treatment induces the expression of ER stress markers but not cfa-miR-346 in a canine macrophage-like cell line

Previously it has been shown that *L.* (*L*.) *infantum* could induce a mild but significant increase in ER stress expression markers, including the spliced form of XBP1 (sXBP1), in human and murine infected macrophages [11].

**Fig. 1:**
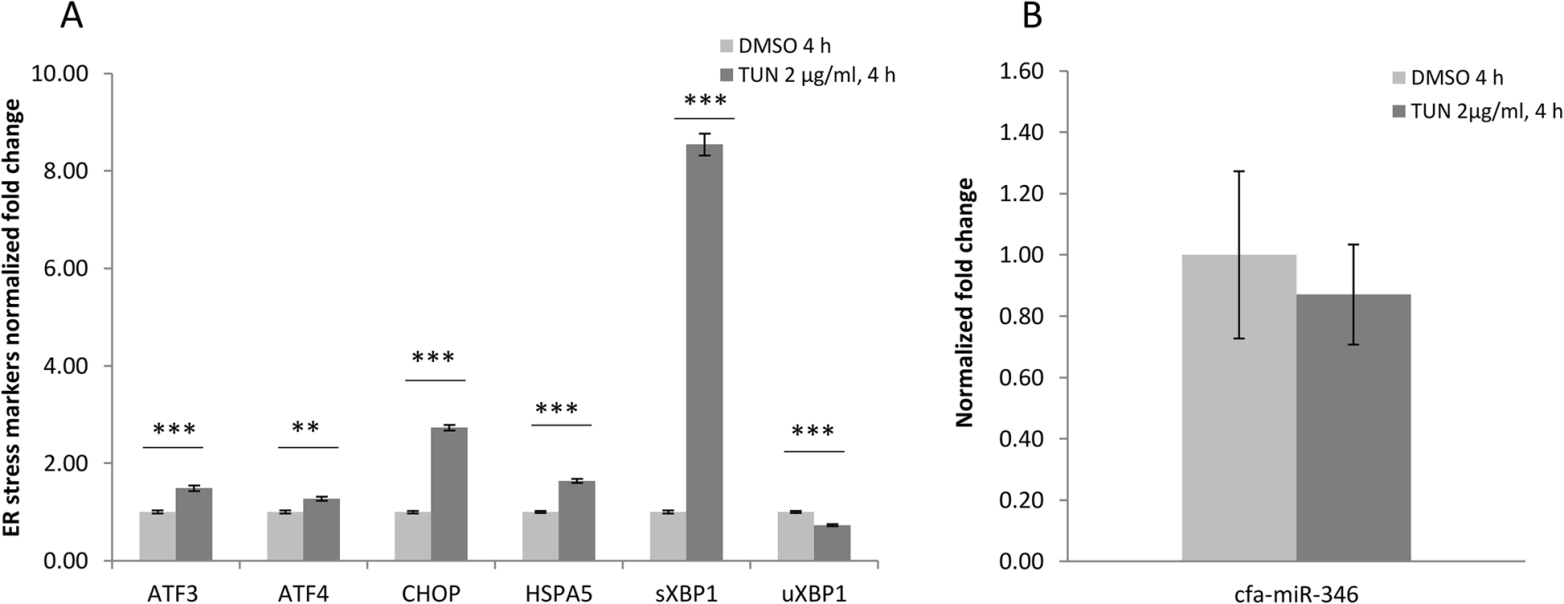
Tunicamycin treatment induces ER stress markers but not cfa-mir-346 in DH82 cells. The ER stress expression markers resulted upregulated in DH82 cells treated with tunicamycin 2 µg/ml for 4 h (**A**), while the expression of cfa-miR-346 did not change significantly (**B**). Data are represented as the mean ± SD of technical replicates and are representative of two independent experiments. The graph shows the fold changes in comparison to the control (DMSO). Unpaired t-test with Welch's correction. *p < 0.05 **p < 0.01 ***p < 0.001

To further explore these pathways in a canine cell line, we first determined whether cfa-miR-346 could be induced following ER stress in DH82 cells treated with tunicamycin, an inducer of ER stress through the inhibition of protein N-linked glycosylation. After 4 h treatment, the gene expression analysis revealed a significant induction of the ER stress markers (i.e., ATF3, ATF4, CHOP, HSPA5, sXBP1) compared to vehicle DMSO (Unpaired t-test with Welch's correction *p* < 0.05) (Fig. 1A). Notably, the unspliced form of XBP1 (uXBP1) resulted downregulated, confirming previous findings in human and murine models. On the contrary, the expression of cfa-miR-346 did not change significantly in response to treatment with Tunicamycin, suggesting a lack of correlation between the upregulation of sXBP1 and the induction of cfa-miR-346 at least at the time point tested (Fig. 1B).

### ER stress response induction by *Leishmania* infection in canine macrophage-like cell line

The ER stress response and induction of cfa-miR-346 were investigated in DH82 cells infected with promastigotes of four *L.* (*L.*) *infantum* strains (*i.e.*, MHOM/TN/80/IPT1, MHOM/FR/78/LEM75, MHOM/IT/08/31U and the canine clinical isolate 42) and a *L.* (*V.*) *braziliensis* human clinical isolate (*i.e*., AN1) for 24 h and 48 h, as described in methods. The infection indexes are reported in Table 1.

**Table 1:** Infection indexes in DH82 cells

*Leishmania* strain/isolate	Infection index^a^	Infection index^a^
	**24 h**	**48 h**
*L.* (*L.*) *infantum* MHOM/TN/80/IPT1	143.3 ± 31.1	168.9 ± 58.8
*L.* (*L.*) *infantum* MHOM/FR/78/LEM75	100.2 ± 22.1	92.7 ± 29.7
*L.* (*L.*) *infantum* MHOM/IT/08/31U	130.0 ± 10.1	167.8 ± 25.8
*L.* (*V.*) *braziliensis* human clinical isolate AN1	185.2 ± 31.2	94.0 ± 26.3
*L.* (*L.*) *infantum* canine clinical isolate 42	189.9 ± 31.9	245.7 ± 50.5

^a^calculated by multiplying the percentage of infected macrophages by the average number of parasites per macrophage**,** means ± SD)

**Fig. 2:**
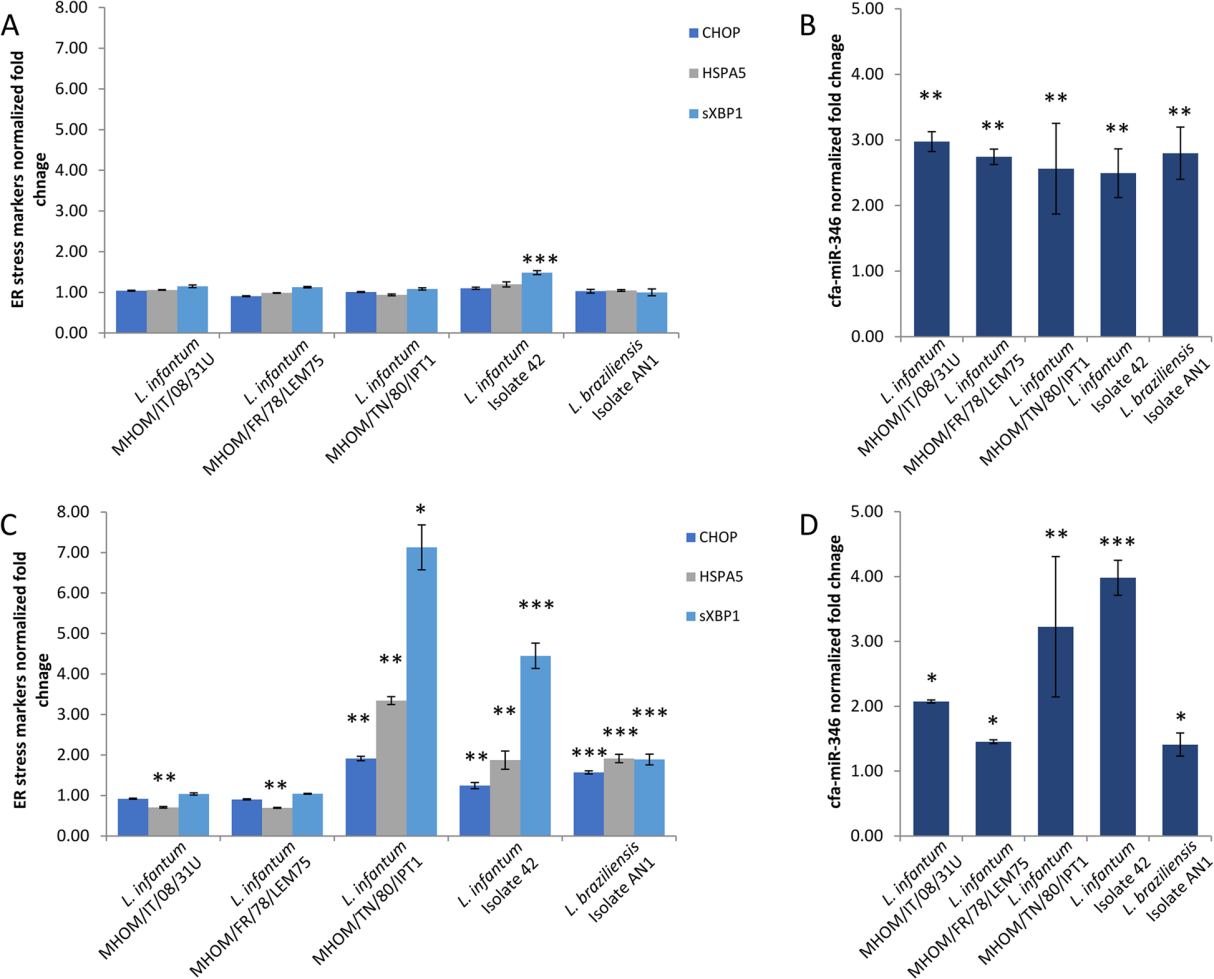
ER stress markers and mir-346 expression in infected DH82 cells. Gene expression of selected ER stress markers and cfa-miR-346 in DH82 cells infected with four *L*. (*L*.) *infantum* strains and one *L*. (*V*.) *braziliensis* strain after 24 h (**A, B**) and 48 h (**C, D**). Graphs show the fold changes related to the control (non-infected cells). Unpaired t-test with Welch's correction * *p* < 0.05 ** *p* < 0.01

A significant induction of ER stress expression markers -including sXBP1- was evident after 48 h infection with *L. braziliensis*, *L. infantum* MHOM/TN/80/IPT1 and isolate 42. However, a significant upregulation of cfa-miR-346 was detected at both 24 and 48 h of infection with all *Leishmania* strains (Unpaired t-test with Welch's correction *p* < 0.05.), suggesting the hypothesis that, in the canine cell model, the induction of cfa-miR-346 could not be related to the induction of ER stress expression markers (Fig. 2).

### Cfa-miR-346 induction is related to *Leishmania* spp. infection, and it is independent from GRID1 expression

To deeply analyze the induction of miR-346 following the infection, and therefore to exclude that its regulation was not related to the phagocytosis mechanism of the macrophage, the infection was performed with both live and heat-killed (HK) parasites as described in methods.

**Fig. 3:**
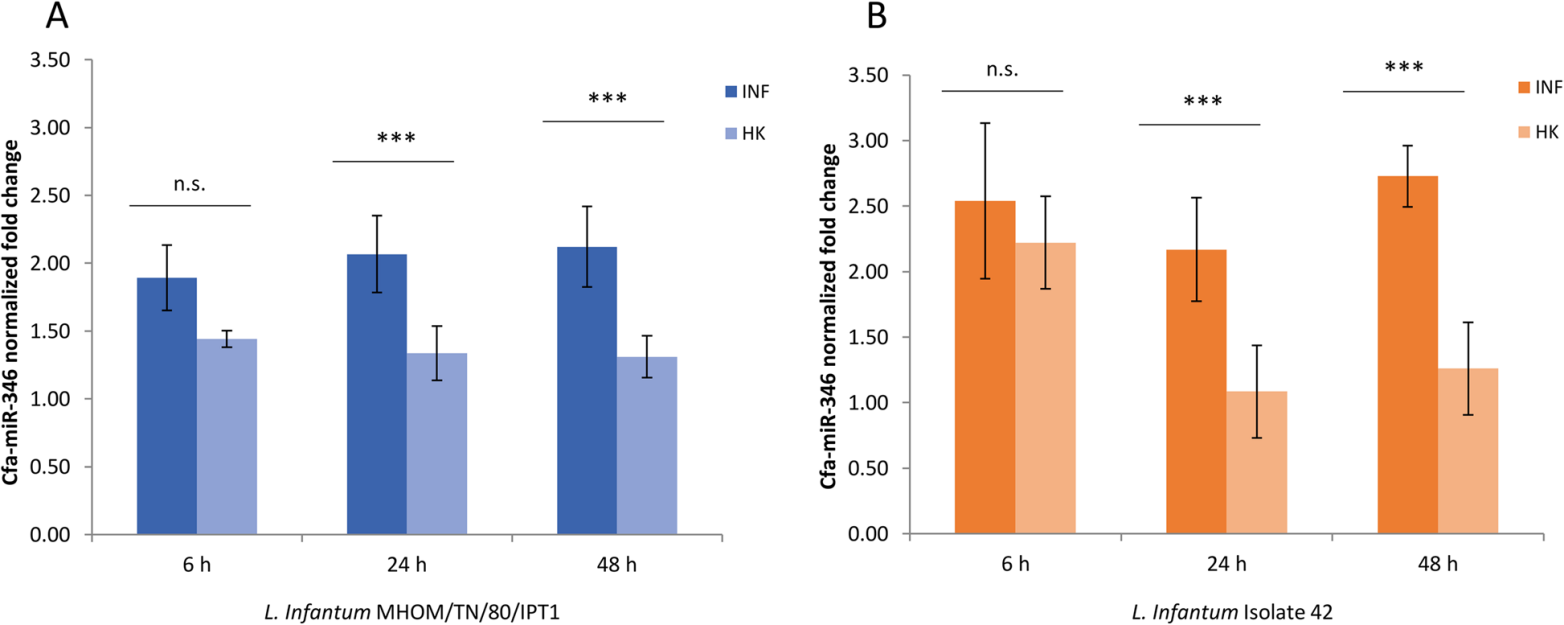
mir-346 induction is related to *Leishmania* infection. cfa-miR-346 expression in DH82 cells infected with *L*. (*L*.) *infantum* MHOM/TN/80/IPT1 (**A**) and canine clinical isolate 42 (**B**) for 6 h, 24 h, 48 h. The graph shows the fold changes in comparison to non-infected cells (mean ± SD of two independent experiments). One-way ANOVA with Tukey’s Multiple Comparison Test. n.s.: not significant, **p < 0.01, ***p < 0.001

A significant induction of miR-346 was evident at 6 h, 24 h, and 48 h in cells infected with live parasites (One-way ANOVA with Tukey's Multiple Comparison Test. *p* < 0.05). After 6 h the induction of mir346 was not significantly different between cells infected with live or HK parasites, probably due to an initial response mediated by macrophage phagocytosis [19, 20]; however, at 24 and 48 h the mir346 expression in cells treated with HK parasites was significantly lower, dropping close to the values of non-infected controls (Fig. 3). These results further confirm that the induction of miR-346 at 24 h and 48 h post-infection is related to the interaction with the vital pathogen.

Cfa-miR-346 is located in intron 2 of the glutamate ionotropic receptor delta type subunit 1 (GRID1) gene. To investigate if mir-346 upregulation could be due to induction of GRID1, the expression level of GRID1 was evaluated by real-time PCR in infected and non-infected DH82 cells. The real-time PCR assays showed no amplification or amplification with Ct > 33 in some technical replicates, leading to the conclusion that GRID1 gene was not expressed in either infected or non-infected DH82 cells (data not shown).

### Cfa-miR-346 expression in plasma of infected dogs

**Fig. 4:**
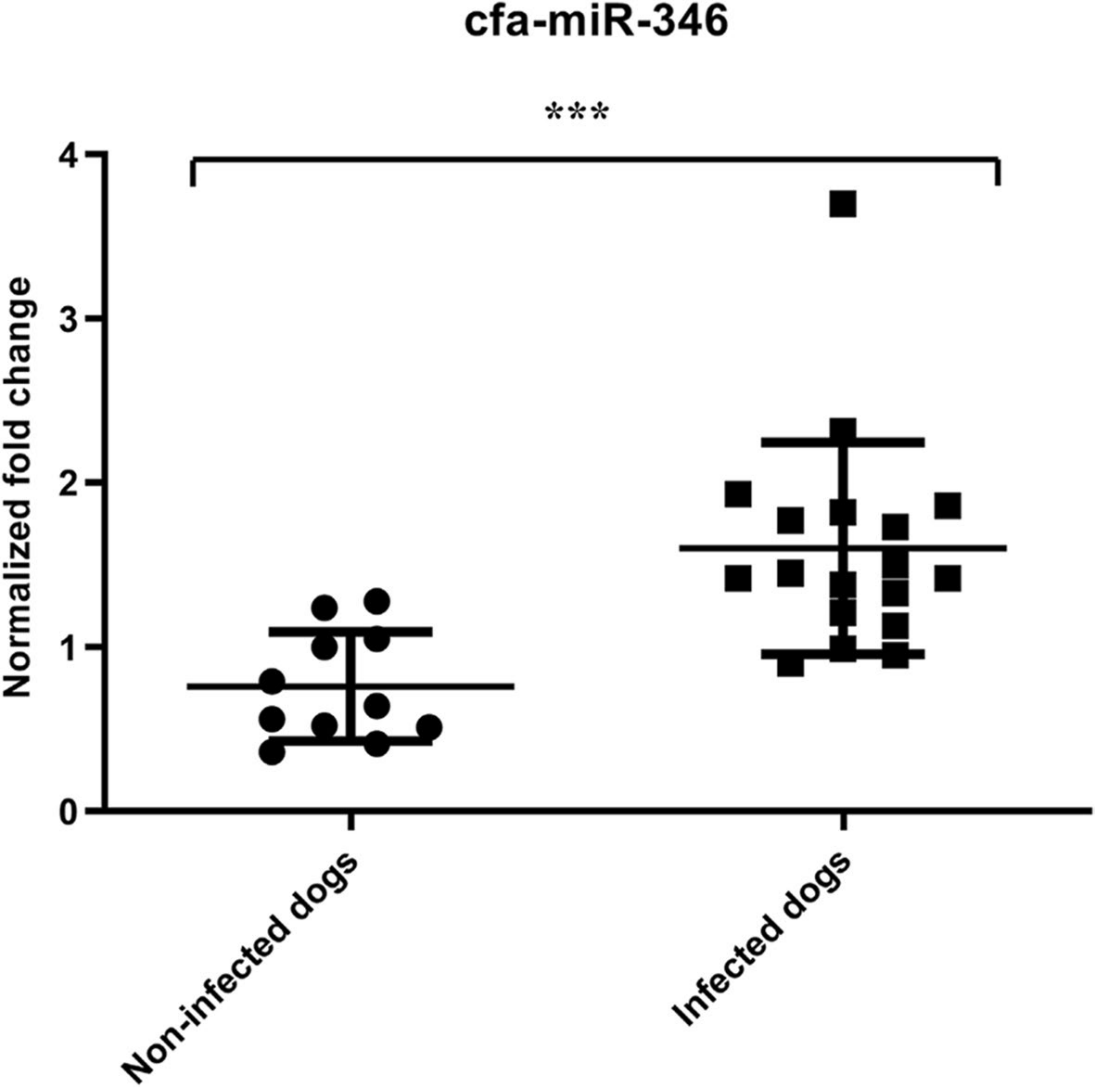
Relative amount of cfa-miR-346 in plasma of non-infected (*n* = 11) and infected dogs (*n* = 18). The scatter plot graph indicates the mean ± SD. Unpaired t-test with Welch's correction. ***p < 0.001

To explore the role of miR-346 in dogs naturally infected with *Leishmania* spp., a study was conducted on 29 mixed breed dogs from Pantelleria island and Marche region (central Italy), both endemic areas for leishmaniasis. All plasma samples used in this study were collected for diagnostic purposes during routine examination. Each dog was subjected to a physical examination to assess the presence of clinical signs compatible with leishmaniasis, to a blood sampling and/or to a lymph node aspirate. The diagnosis of CanL was based on the presence of clinical signs (weight loss, skin, and eye signs, lymphadenomegaly), serological and/or qPCR-based methods, performed on blood and/or lymph node aspirates. In two cases (dog IDs 42 and 64) the isolation of the parasite was also obtained. Following the diagnostics evaluation, the dogs were divided into two groups, negative and positive, for *Leishmania* infection, and the relative expression of miR-346 was evaluated in plasma samples obtained from all dogs. Despite the partial overlap between the distribution of miR-346 expression, the results showed that the levels of miR-346 in plasma of dogs naturally infected with *L*. (*L*.) *infantum* is significantly higher compared to non-infected dogs, confirming the previous finding obtained in vitro and suggesting the induction of miR-346 following active infection (Fig. 4).

## Discussion

Many different mechanisms activated by *Leishmania* spp*.* to survive within the host cell have been described, e.g. counteracting the antigen presentation and interfering with the macrophage signaling cascade [10]. Among them, the role of the ER stress and Unfolded Protein Response (UPR), including the activation of IRE1-XBP1 and PERK/eIF2α/ATF4 arms of UPR, have also been investigated [11, 12, 21].

Although dogs are considered of primary importance in the epidemiology of leishmaniasis, the majority of the current knowledge about *Leishmania*-macrophage interaction comes from cell cultures of murine or human origin. In fact, a limited number of works have been conducted using the canine macrophage-like cell line DH82 as a cell model to study *Leishmania* infection. The DH82 cells can be considered an M2 subtype macrophage model for leishmaniasis [22], however, these cells were less permissive to *L. infantum* infection and showed a lower number of amastigotes per macrophages compared to mouse macrophages or human U937 cell line [23]. This was evident also in the present work, since the infection indexes were rather low compared to human or murine models used in our previous publications, where infection indexes were approximately 150–600 [11, 16].

This could be explained by the fact that DH82 cells were not activated by PMA prior to the addition of the parasites; therefore, DH82 cells can multiply during the infection, influencing the infection index. Moreover, little is known about the surface molecules presented in these cells involved in parasite recognition and uptake. The low infection index could explain the late induction of ER stress markers, which was evident only at 48 h post-infection, in particular with *L. infantum* MHOM/TN/80/IPT1 and canine clinical isolate 42.

A number of recent studies demonstrated that infections caused by several *Leishmania* species can induce alteration of miRNA profile in human, murine, and canine macrophages [17]. We have recently investigated mir-346 expression in human U937 and THP-1-derived macrophages infected by *L. infantum* or *L. braziliensis*, finding a significant upregulation of this miRNA during infection [16].

In this work, we extended these findings to a canine macrophage-like cell line (DH82) by analyzing the induction of cfa-miR-346 after ER stress induction or *Leishmania* infection.

First, we showed that treatment of DH82 cells with tunicamycin significantly induced ER stress markers (including sXBP1); however, the mir-346 expression did not change at the time point tested, accounting for a sXBP1-independent mechanism of mir-346 induction in this cell line, contrary to what happened in humans [15, 16]. Although the phylogenetic relatedness between humans and dogs, the pathogenesis and the clinical manifestation of the disease is different, probably related also to a Th1/Th2 alternative activation and balance. In fact, while dogs with a chronic disease show a prevalence of Th2 response, there is not a clear Th1/Th2 dichotomy in the T-cell response in human leishmaniasis [24]. Moreover, a recent work evidenced the variability of immune response between humans and dogs related to differences in complement proteins amount [25], which could play an important role in parasite inactivation due to the different lytic properties, affecting the clinical manifestations [26]. Nevertheless, the differences in immune responses related to ER stress and miRNA dysregulation have not been investigated yet. It is noteworthy, the ER stress markers induction with tunicamycin resulted mild compared to U937, THP1 or murine macrophages (in which for example, sXBP1 was 20–100 fold upregulated) [11, 16]. In the same way, Nadaes et al. showed general low responsiveness in DH82 stimulated by LPS compared with RAW264.7 cells in terms of reactive oxygen species (ROS) and nitric oxide (NO) production, underling a different activation mechanism in this cell line [22]. A significant induction of ER stress expression markers -including sXBP1 was observed after 48 h post-infection. Similar results have been obtained from the transcriptome analysis in dogs naturally infected where an overexpression of XBP1 and some other genes involved in ER stress and UPR have been evidenced in lymph nodes [27]. On the other hand, the cfa-mir-346 was always significantly upregulated after infection with all *Leishmania* strains and at all times tested, including 24 h from infection, when the expression of ER stress markers was still unchanged. Notably, we also showed that induction of cfa-mir-346 at early time of infection (6 h) can be elicited also by HK parasites, probably be due to an initial response to macrophage-mediated phagocytosis; however, cfa-miR-346 upregulation was maintained at later time points (24 h and 48 h) only in cells infected with viable parasites. Taken together, these data account for a mechanism of cfa-mir-346 induction in canine macrophage-like cells i) depending on infection with live parasites and ii) not necessarily related to the induction of the ER stress marker sXBP1.

Afterward, we investigated the presence of cfa-miR-346 in the plasma of dogs naturally infected by *L. infantum* in comparison with plasma obtained from non-infected dogs. Our data showed a significant increase of the cfa-miR-346 expression levels in the plasma of infected dogs, suggesting a possible application of the circulating cfa-mir-346 as low-invasive marker of infection. Nevertheless, the partial overlap of miR-346 expression levels between the two groups, probably due to inter-individual variability among the subjects involved in the study, did not allow to propose this miRNA as a diagnostic tool for leishmaniasis. In fact, canine leishmaniasis is characterized by different clinical manifestations and stages of the disease and different levels of severity. Previous works showed that hsa-miR-346 was correlated with various diseases in humans and it has been indicated as a potential biomarker of disease progression and therapeutic target [28–30]. In dogs, cfa-miR-346 resulted upregulated in cardiac hypertrophy [31]. Regarding the infectious diseases in humans, similar to our findings, Nishimura et al*.* showed that hsa-miR-346 is secreted from macrophages infected by the intracellular pathogen *Mycobacterium avium* complex -which has been demonstrated to elicit ER stress-induced apoptosis in infected macrophages [32], and proposed that its serum levels could be a potential biomarker of *Mycobacterium avium* complex pulmonary disease activity [33]. In the same way, hsa-miR-346 was found to be a potential marker in monitoring the efficacy of treatments against *Mycobacterium tuberculosis* in patients with tuberculosis [34]. Since the expression of miR-346 can be influenced by various diseases, in both humans and dogs, further clinical studies will be necessary to identify the increase of cfa-miR-346 expression as a specific marker of CanL and exclude other infectious diseases that are common in dogs.

## Conclusion

In summary, miR-346 was found to be upregulated in the canine macrophage-like cell line DH82 infected with four different strains/isolates of *L. (L.) infantum*, as well as one *L. (V*.) *braziliensis* isolate, confirming previous findings in human cell lines and pointing to a possible common pathogenic mechanism in human and canine host cells. Moreover, miR-346 has been shown to be upregulated in the plasma of dogs naturally infected by *L. infantum*. Nevertheless, miR-346 dysregulation is associated with various diseases, infectious or non-infectious, in both humans and dogs. According to our results, an increase of cfa-miR-346 plasma level could represent a promising potential biomarker of leishmaniasis, even though further studies will be needed to understand its role during *Leishmania* infection and to evaluate its specificity as a possible biomarker of canine leishmaniasis.

## Methods

### *Leishmania* parasites cultivation

Three *L.* (*L*.) *infantum* reference strains (MHOM/TN/80/IPT1, MHOM/FR/78/LEM75 and MHOM/IT/08/31U), and one *L.* (*L.*) *infantum* canine clinical isolate (isolate 42) were provided by the OIE Reference Laboratory National Reference Centre for Leishmaniasis (C.Re.Na.L.) (Palermo, Italy). Moreover, a *L.* (*V.*) *braziliensis* clinical isolate (isolate AN1), identified at the species level by PCR–RFLP as described by Schönian et al. [35], was obtained from a pharyngolaryngeal biopsy during routine diagnosis of a human patient with suspect MCL, as previously described [16, 36]. *Leishmania* promastigotes were cultivated at 26–28 °C in Evans’ Modified Tobie’s Medium (EMTM) and/or RPMI-PY medium [37], and stationary promastigotes were transferred to fresh medium (ratio 1:5) every 5–7 days.

### Cell culture and infection

The canine macrophage-like cell line DH82 (ATCC® CRL-10389™) was cultured in an incubator at 37 °C and 5% CO_2_ in Eagle’s Minimum Essential Medium (EMEM) supplemented with 15% heat-inactivated Fetal Bovine Serum (FBS), 2 mM L-glutamine, 10 g/l Non-Essential Amino Acid, 1 mM sodium pyruvate, 100 μg/ml streptomycin, 100 U/l penicillin. For infection experiments, 2.5 × 10^5^ cells were seeded in 35 mm dishes for 24 h for cell adhesion. Then, cells were infected with stationary-phase promastigotes of *L.* (*L*.) *infantum* strains/isolate or *L.* (*V.*) *braziliensis* clinical isolate with a parasite-to-cell ratio of 10:1. To exclude the modulation of target genes by the phagocytosis mechanism of macrophages, 2.5 × 10^6^ promastigotes of *L.* (*L.*) *infantum* MHOM/TN/80/IPT1 and canine clinical isolate 42 were inactivated (heat-killed, HK) at 70 °C for 45 min and placed into DH82 culture medium. To promote and synchronize cell infection, dishes were centrifuged at 450 × g for 3 min at room temperature. After 24 h, non-internalized parasites were removed by medium replacement. As ER-stress positive control, cells were treated with 2 μg/ml of tunicamycin for 4 h or with vehicle dimethyl sulfoxide (DMSO) as control. All cell culture reagents were purchased from Sigma-Aldrich (St. Louis, MO). After 6 h, 24 h, and 48 h from infection, cells were directly lysed for gene expression analysis by adding QIAzol Lysis Reagent to the cell-culture dish as described below in the RNA isolation and cDNA synthesis section. Moreover, one dish for each time point was stained with Hoechst and Acridine Orange dyes, and the infection index was calculated by multiplying the percentage of infected macrophages by the average number of parasites per macrophage. At least 300 macrophages were counted for each condition.

### Study design

No direct intervention was done on animals. All plasma samples used in this study were surplus material collected for diagnostic purposes during routine examination; oral informed consent was obtained from the owners of dogs.

To evaluate the presence of cfa-miR-346 in clinical samples of non-infected and infected dogs, 21 plasma samples of mixed breed dogs from Pantelleria Island (Sicily, Italy), an endemic area for leishmaniasis, have been obtained by the C.Re.Na.L. in Palermo (Italy) [38]. Additional 8 plasma samples have been supplied by the veterinary clinic “Santa Teresa” in Fano (Pesaro-Urbino, Italy). To diagnose CanL, every dog was subjected to physical, clinical, and dermatological evaluation, including the anamnestic history, changes compatible with CanL (e.g., alopecia and desquamation, nodular or ulcerative lesions, exfoliative dermatitis weight loss, lymphadenomegaly). In addition, serological [i.e. IFAT, SNAP test (IDEXX Laboratories, Westbrook, Maine, USA) or Speed Leish K test (BVT Groupe Virbac, La Seyne sur Mer, France] and qPCR-based tests [39] were performed on blood and/or lymph nodal aspirates. In two cases (IDs 42 and 64), *L.* (*L.*) *infantum* amastigotes isolation was obtained as previously described [40]. After diagnosis, dogs were divided into two groups: i) non-infected: negative for clinical signs and/or negative IFAT, negative qPCR, negative for SNAP test or Speed Leish K test; ii) infected: presence of clinical signs, IFAT ≥ 1:40 and/or positive qPCR, and/or positive SNAP test or Speed Leish K test [41]. Details of samples are summarized in Table 2.

**Table 2:** Canine sample ID, summary of clinical signs, and results of diagnostic methods

Sample ID	Clinical signs	IFAT	SNAP TEST	qPCR (blood)	qPCR (Lymph node)	Parasite isolation	Diagnosis of leishmaniasis
24	Lymphadenomegaly	Neg	n.a	Neg	Neg	n.a	-
29	Neg	Neg	n.a	Neg	n.a	n.a	-
35	Neg	Neg	n.a	Neg	Neg	n.a	-
43	Eye signs	Neg	n.a	Neg	n.a	n.a	-
46	Neg	Neg	n.a	Neg	n.a	n.a	-
52	Neg	Neg	n.a	Neg	n.a	n.a	-
54	Neg	Neg	n.a	Neg	n.a	n.a	-
GOA	Neg	Neg	n.a	n.a	n.a	n.a	-
GON	Neg	Neg	n.a	n.a	n.a	n.a	-
VIA	Neg	Neg	n.a	n.a	n.a	n.a	-
ZAR	Neg	Neg	n.a	n.a	n.a	n.a	-
9	Lymphadenomegaly	1:160	n.a	Neg	Pos	n.a	+
11	Lymphadenomegaly	1:320	n.a	Neg	n.a	n.a	+
23	Skin signs, Lymphadenomegaly	1:320	n.a	Neg	Pos	n.a	+
30	Skin signs, Lymphadenomegaly	1:320	n.a	Neg	Neg	Bacterial contamination	+
31	Skin signs	1:80	n.a	Neg	Pos	n.a	+
32	Neg	1:320	n.a	Neg	Neg	n.a	+
40	Skin signs	1:320	n.a	Neg	Pos	n.a	+
42	Neg	1:5120	n.a	Neg	Pos	Pos	+
45	Neg	1:40	n.a	Neg	Pos	Bacterial contamination	+
49	Eye signs	1:160	n.a	Neg	n.a	n.a	+
51	Neg	Neg	n.a	Neg	Pos	n.a	+
58	Lymphadenomegaly	1:2560	n.a	Neg	Pos	n.a	+
63	Neg	1:80	n.a	Neg	Pos	n.a	+
64	Skin and eye signs, lymphadenomegaly	1:2560	n.a	Neg	Pos	Pos	+
STA-VOI	Mild periocular dermatisis, anemia, thrombocytopenia	n.a	Pos	n.a	n.a	n.a	+
MAI-EUI	Alopecia, weight loss, mild onychogryphosis, lymphadenomegal, leucopenia, decline in performance	n.a	Pos	n.a	n.a	n.a	+
MIA-MAI	Chronic anemia	n.a	Pos	n.a	n.a	n.a	+
KIO-MEI	Neg	n.a	Pos	n.a	n.a	n.a	+

*POS* Positive *NEG* Negative, *NA* Data not available

### Plasma collection

One mL of peripheral blood was collected from the jugular vein of each dog and placed into tubes containing ethylene diamine tetraacetic acid (EDTA). Blood was centrifuged at 200 × g for 15 min at 4 °C to separate plasma from the corpuscular part. Plasma collected was further centrifuged at 800 × g for 15 min at 4 °C to remove any cellular debris. The supernatant (150 µl) was transferred in a sterile tube and processed as described below.

### RNA isolation and cDNA synthesis

RNA extraction from DH82 infected and non-infected cells was performed with the miRNeasy Mini Kit (Qiagen, Hilden, Germany) after direct lysis with 700 µl of QIAzol Lysis Reagent (Qiagen, Hilden, Germany). RNA extraction from plasma was carried out with Total RNA Purification Kit (Norgen Biotek Corporation, Ontario, Canada) according to the manual instructions specific for plasma. In both cases, extracted RNA was quantified with a NanoVue PlusTM spectrophotometer (GE Healthcare Life Sciences, Piscataway, NJ, United States).

For gene expression analysis in DH82 cells, total RNA (500 ng) was reverse-transcribed using PrimeScriptTM RT Master Mix (Perfect Real Time) (Takara Bio Inc.). For expression analysis of miR-16 and miR-346 in all samples, total RNA (1–5 ng/μl) was reverse-transcribed with the TaqMan™ MicroRNA Reverse Transcription Kit (Applied Biosystems).

### Quantitative real-time PCR

The expression of several ER stress markers was evaluated by qPCR as previously described [11] with some modifications. Briefly, the qPCR reactions were carried out in duplicate in a final volume of 20 µl using TB Green PreMix ex Taq II Master Mix (Takara Bio Europe, France) and 200 nM primers listed in Table 3. The amplification conditions were: 95 °C for 10 min, 40 cycles at 95 °C for 10 s and 60 °C for 50 s, followed by a melting curve analysis at the end of each run from 65–95 °C, to exclude the presence of non-specific products or primer dimers. As reference gene, GAPDH (glyceraldehydes-3-phosphate dehydrogenase) was selected among three candidates (B2M, GAPDH, GUSB). The expression of GRID1 was evaluated with primers GRID1_F and GRID1_R (Table 3), using the same conditions described above. All primer pairs targeting canine genes were designed using Primer-BLAST.

**Table 3:** Primers used in this study

Target mRNA	Accession number	Forward primer (5’-3’)	Reverse primer (5’-3’)
Atf3	XM_022420880.1	TTCGCCATCCAGAACAAGCA	GGGCTACCTCAGTTTTCGTG
Atf4	XM_854584.5	TTCTCCAGCGACAAGGCTAA	AAGGCATCCTCCTTTCCGTTG
Ddit3 (Chop)	XM_014117187.2	CTGGAAACAAGGAGGAAGAATCA	GGCTCTGGAAGGTGTTCGTG
Grid1	XM_022417615.1	TACAGCAAGGTGGCGAATCCT	AGGAGCACACAATGAGGGTGA
Hspa5	XM_858292.5	TGGCATAAACCCAGACGAGG	AGGGGACATACATCAAGCAGT
sXbp1	XM_849540.5	CTGAGTCCGCAGCAGGT	TGAACAGAATGCCCAACAGG
uXbp1	XM_849540.5	CCGCAGCACTCAGACTACG	TGAACAGAATGCCCAACAGG
Gapdh	NM_001003142.2	GTCCCCACCCCCAATGTATC	TCCGATGCCTGCTTCACTAC

*Atf3* Canis lupus familiaris activating transcription factor 3, *Atf4* Canis lupus familiaris activating transcription factor 4, *Ddit3* Canis lupus familiaris DNA damage inducible transcript 3, *Grid1* Canis lupus familiaris glutamate ionotropic receptor delta type subunit 1, *Hspa5* Canis lupus familiaris heat shock protein family A (Hsp70) member 5, *sXbp1* Canis lupus familiaris X-box binding protein 1 (spliced), *uXbp1* Canis lupus familiaris X-box binding protein 1 (unspliced), *Gapdh* Canis lupus familiaris glyceraldehyde-3-phosphate dehydrogenase

To evaluate the expression of cfa-miR-346, the qPCR reactions were performed with a specific Taqman small RNA assay (Applied Biosystems), according to the manufacturer instructions. The amplification protocol was: 95 °C for 10 min, 50 cycles at 95 °C for 15 s and 60 °C for 50 s. The microRNA cfa-miR-16 was used as reference gene [42, 43]. All qPCR reactions were performed in a RotorGene 6000 instrument (Corbett life science, Sydney, Australia). A duplicate non-template control was included for each primer pair reaction as negative control. The relative expression levels were calculated using the 2^−ΔΔCt^ method [44].

### Statistical analysis

Statistical analysis was performed by unpaired t-test with Welch's correction or one-way ANOVA with Tukey’s multiple-comparison post-test. Values are expressed as mean ± standard deviation (SD). All statistical tests were performed using GraphPad Prism version 8.02 (GraphPad Software, Inc., La Jolla, CA, USA). A *P* value ≤ 0.05 was considered statistically significant.

## Data Availability

The datasets used and/or analyzed during the current study are available from the corresponding author on reasonable request.

## Abbreviations

WHO: World Health Organization
VL: Visceral leishmaniasis
CL: Cutaneous leishmaniasis
MCL: Mucocutaneous leishmaniasis
PCR–RFLP: Polymerase chain reaction-Restriction Fragment Length Polymorphism
qPCR: Quantitative real-time PCR
BLAST: Basic local alignment search tool
IFAT: Immunofluorescence antibody test
